# Exploring the Relationship Between Social Commerce Features and Consumers’ Repurchase Intentions: The Mediating Role of Perceived Value

**DOI:** 10.3389/fpsyg.2021.775056

**Published:** 2022-03-25

**Authors:** Jinyuan Guo, Lei Li

**Affiliations:** ^1^College of Economics and Management, South China Agricultural University, Guangzhou, China; ^2^College of Economics and Management, Northwest Agriculture and Forestry University, Yangling, China

**Keywords:** social commerce, interactivity, recommendations, feedback, utilitarian value, hedonic value

## Abstract

The popularity of social media, such as WeChat and Weibo in China, has provided an opportunity to develop social commerce. Although shopping through social commerce platforms is widely favored by consumers, the factors affecting consumers’ decision-making behavior in the social commerce environment remain unclear. Therefore, from the perspective of the stimulus–organism–response (SOR) theory, we construct a consumer repurchase decision model in the social commerce environment and analyze the influencing mechanism of social commerce features (interactivity, recommendations, and feedback) on perceived value (utilitarian value and hedonic value) and consumers’ repurchase intention. The empirical results found that social commerce features are positively related to the generation of perceived value, which in turn drives consumers to form repurchase intentions. We also found some mediating effects of perceived value. The study’s conclusions clarify the intrinsic influence mechanism of social commerce features on consumers’ perceived value and repurchase intentions. In addition, it can provide some theoretical guidance for future research and business.

## Introduction

With the progress of information technology, consumers have had increasing access to social media. From email to Weibo and WeChat, social media constantly meets users’ needs for social interaction (Hajli, 2014). Statistics show that as of December 2020, there are nearly one billion Internet users in China, and the number of users of instant messaging applications (such as WeChat) has reached 981 million, accounting for 99.2% of the total Internet users (CNNIC, 2021). Social media is becoming the primary method by which people communicate and transmit information in daily life, and its ability to connect users with various life services is constantly expanding (Bowen et al., 2021). It has changed the way people communicate, cooperates, lives, and operate businesses and promoted the emergence and development of a new e-commerce model—social commerce (Busalim and Hussin, 2016). Social commerce is an online business application that guides users to participate in the online market and community through social media and Web 2.0 technology to promote the sale, purchase, and information sharing of products and services in the community (Zhou et al., 2013). By aggregating and analyzing a series of user-generated content such as text, pictures, and videos, social commerce enables companies to reach and understand a much more comprehensive range of consumers more efficiently than traditional offline stores (Yan et al., 2021). With social media as a link, companies can narrow the distance with customers and establish higher-quality customer relationships, thereby increasing consumer satisfaction and loyalty to the company (Hajli, 2014). Through the significant impact on communication efficiency, user experience, user relationship quality, and corporate marketing models, social commerce can significantly enhance a company’s competitive advantage in the new sales environment (Shin and Lee, 2021). Additionally, customers in social commerce can use online collaboration to exchange important product information and personal experience and obtain valuable suggestions from other community members to make more intelligent and more accurate purchasing decisions (Priharsari and Abedin, 2021). Therefore, the development of social commerce plays an irreplaceable role in enterprises and the lives of consumers.

The rise and prosperity of social commerce have also attracted attention from the academic community (Tiwary et al., 2021). Social commerce is considered to be an extension of e-commerce, but it is different from e-commerce (Han et al., 2018). As shown in Table 1, the scope, business objectives, customer connection, and system interaction of social commerce differ from e-commerce (Mou and Benyoucef, 2021). Social commerce mainly emphasizes social activities through social media, connecting consumers who act alone in e-commerce, enhancing communication and dialog between them, expressing their opinions, and sharing helpful information with others (customers and enterprises; Han et al., 2018). Furthermore, consumers’ roles can be flexibly converted and traded freely. Baghdadi (2013) noted that these differences are reflected in, for example, business models, value creation, customer contact and communication, system interaction, platform design, and technical foundations. Similarly, Lee et al. (2013) explained the differences between social commerce in core concepts, motivations for change, rationality standards, business platforms, trading mechanisms, and principal agents.

**Table 1:** The difference between e-commerce and social commerce.

	E-commerce	Social commerce	References
Scope	Product-centric and users search and purchase products online based on the information provided by the company	Customer-centric and companies provide online communities that support social connections to motivate users to shop	Li and Ku, 2018
Business objective	Through associated search, simplifying purchases, and recommendation strategies based on consumption records to maximize consumer stickiness and shopping efficiency	Aims to achieve social goals, establish a relationship network with customers, share information, and promote member collaboration and value co-creation	Meilatinova, 2021
Customer connection	Customers usually interact with e-commerce platforms independently of other customers	Customers join an online community that supports social connections and encourage conversations between customers and customers and between customers and sellers	Li and Ku, 2018
System interaction	One-way browsing is almost always provided, where little information from the customer is fed back to the business or other customers	More social, interactive, and collaborative ways have been developed to enable customers to fully express themselves and share information with other customers and businesses	Huang and Benyoucef, 2015

Value significantly drives individual behavior, and when people perceive value from a particular behavior based on their experience or knowledge, they form a positive attitude toward the action (Chen, 2017). In the social commerce environment, perceived value is a subjective perception of consumers’ interaction process and results, which is closely related to their social experience (Tiwary et al., 2021). Through perceived value, we can have a deeper understanding of the specific impact of social commerce features on consumers, and the difference in the weight of features, to clarify the focus of consumers’ attention (Hu et al., 2016). At this point, merchants can identify key social commerce features based on their impact differences and enhance these features to build more inclusive communities that meet the needs of different consumers (Xi and Hamari, 2020). In addition, with the emergence of personalized needs, consumers pay more attention to the realization of social goals in social commerce, such as information sharing, emotional expression, and social interaction (Meilatinova, 2021). The more these goals are achieved, the more perceived value consumers get. Because of this, the perceived value of consumers more clearly reflects the attractiveness of social commerce platforms to them, and it can more strongly predict whether consumers are willing to continue participating in the platform. Previous studies have also shown that perceived value is the strongest predictor of purchase intention in online shopping, significantly promoting consumers’ repeated and continuous purchase behavior (Gupta and Kim, 2010; Liu et al., 2021).

Although social commerce has great development potential and is increasingly favored by consumers, there are still obvious research gaps in the existing literature. First, the perceived value represents the comprehensive impression of social commerce features in consumer groups, which is the key to attracting consumers’ extensive participation in social commerce and an important influence factor that drives consumers to purchase (Bugshan and Attar, 2020; Mou and Benyoucef, 2021). However, it is unclear whether social commerce features affect consumers’ perceived value and the difference of such influence under different value dimensions, which hinders the exploration of subsequent consumer behavior (Aspara et al., 2021). Therefore, we need to conduct a detailed and in-depth analysis to clarify the mediating role of perceived value to reveal the influence mechanism of social commerce features on consumers’ purchase intention. Second, existing studies pay little attention to consumers’ repurchase intention and still focus on purchase intention. They do not realize the importance of repurchase intention to the sustainable development of social commerce platforms and do not fully discuss the factors affecting repurchase intention (Meilatinova, 2021). Therefore, it is urgent to conduct relevant research to clarify the influencing factors and mechanism of consumers’ repurchase intention in social commerce.

Based on the above analysis, from the perspective of perceived value, this study constructs the influencing factors model of consumers’ shopping decisions in the socialized commerce environment and discusses the influencing mechanism of social commerce features on consumers’ repurchase intention. We use survey data from 514 Chinese WeChat users to test the proposed research model, and the data analysis results support all our research hypotheses well. Through theoretical explanation and empirical analysis, this study clarifies the unique characteristics of social commerce, proves that interactivity, recommendations, and feedback play a key role in improving consumers’ perceived value, and expands the previous research on social commerce and perceived value. Next, this study also verified the positive impact of utilitarian value and hedonic value on repurchase intention and emphasized the importance of the two value dimensions in social commerce and how they promote the generation of consumers’ repurchase intention. Meanwhile, our research also provides some guidance for the industry and practitioners. By understanding the core features of social commerce, companies can more purposefully strengthen the functions and quality of social commerce platforms to meet the ever-changing needs of consumers. In addition, companies can also formulate differentiated marketing strategies and product services for different consumers based on the importance of perceived value so that they can perceive more utilitarian value and hedonic value, thereby inspiring their repurchase intention.

## Theoretical Foundations

### Consumer Behaviors in Social Commerce

Social commerce enables consumers to establish digital connections with each other and with commercial entities, which is defined as the “exchange-related activities that occur in, or are influenced by, an individual’s social network in computer-mediated social environments, where the activities correspond to the need recognition, pre-purchase, purchase, and post-purchase stages of a focal exchange” (Yadav et al., 2013, p. 312). This definition emphasizes the exchange activities at all stages of consumer decision making and the meaningful personal connections and continuous social interaction between network members (Zhang and Benyoucef, 2016). Consumer behavior in social commerce, especially purchase decision, has always been a hot topic of IS and market research (Busalim et al., 2021). For example, Mou and Benyoucef (2021) tested the effect of the factors derived from consumer behavior theory on the stages of consumer decision making through meta-analysis. Akram et al. (2021) analyzed the effect of hedonic and utilitarian motivations on online purchase intention under Chinese social commerce environment. Bhattacharyya and Bose (2020) examined how likes on Facebook influence user’s purchase and recommendation decisions on a linked e-commerce website. Sohn and Kim (2020) explored the influence of economy, necessity, reliability, and sales promotion on purchase intentions. Li et al. (2019) investigated the influences of social commerce sites on customers’ virtual experiences and on their intentions to purchase products from the perspective of social interactions. Lin et al. (2017) indicated that social commerce features would influence consumers’ repurchase intention through trust and swift guanxi. Obviously, perceived value and social commerce features have been verified as important antecedents of consumers’ purchasing decisions, but the relationship between them is a black box, and we do not know whether perceived value plays a mediating role between social commerce features and purchase intention. Thus, this study has explored the influence of social commerce features on consumers’ repurchase intention from the perspective of perceived value.

### The Stimulus–Organism–Response Theory

To study the factors that affect individual behavior, Mehrabian and Russell (1974) developed stimulus–organism–response (SOR) theory, which believes that various types of environmental stimuli (S), such as tangible money and intangible time, may influence an organism’s (O) emotion and internal state, including perception, physiology, feelings, and thinking activities, which in turn drives them to make behavioral responses (R), such as satisfaction and purchase intention (Lee and Yun, 2015). In the online shopping environment, environmental stimuli are mostly the website quality and product functions of the shopping platform, while the organism is generally the behavior and emotional changes of consumers, and specific consumption behavior or intention is the response (Zhang et al., 2014). Therefore, this study takes the features of social commerce as the stimuli (S), consumer’s perceived value as the organism (O), and repurchase intentions as the response (R).

Based on previous studies, we found that the SOR theory is suitable for consumer behavior research, and it has also been widely adopted by related research. For example, Li (2017) believes that social commerce affects consumers’ cognitive and emotional states, determining their virtual experience in shopping and corresponding purchase intention. Fu et al. (2018) examined the effects of similarity between members in the social community on social shopping intentions from the perspectives of perceived usefulness (PU), perceived enjoyment (PE), and trust transfer. These studies confirm that the SOR theory helps explain the impact of environmental stimuli on consumers’ internal state and behavioral responses. Through SOR theory, this study can more clearly explore how stimulating factors in social commerce environments affect the internal states of consumers and then prompt them to generate corresponding behavioral responses.

### Social Commerce Features as Environmental Stimuli (S)

Social commerce is a new business model mediated by social media technology, which involves integrating social media and business activities. It includes both enterprise-related and consumer-related activities (Yadav et al., 2013). Enterprise-related activities focus on establishing and maintaining online communities where users can communicate freely, attracting new users through similarities among members and developing them into new sources of user-generated content (Fu et al., 2018). This significantly reflects social commerce features. For example, in social commerce, consumers deliver personalized shopping experiences to others through text, voice, pictures, and videos to provide references and help other users (Lai and Turban, 2008). This process reflects the consumer-related activities in social commerce, which involve generating user-generated content through interactions and information sharing between consumers (Bugshan and Attar, 2020). In addition, enterprises can collect consumers’ feedback through information exchange in the community and use the feedback to improve products and services and promote consumers’ repurchase intentions (Hajli, 2015). Thus, social commerce features have a substantial impact on consumer behavior.

When the SOR theory is applied to the background of social commerce, the stimulus (S) represents the unique features of social commerce. Shadkam and O’Hara (2013) found that these features can enhance the connection between consumers and enable them to share information, recommend to each other, and post comments. Moreover, social commerce is also committed to creating an excellent atmosphere to encourage consumers to actively participate in the interaction of products and services, share information and experience with others, and thereby promote the generation of user content (Horng and Wu, 2020). Through the integration and generalization of user-generated content, social commerce can create commercial value for the platform and enhance consumers’ participation and social support in the shopping process and their confidence in shopping (Hajli, 2014). Hence, an increasing number of scholars have studied the features and structure of social commerce to more deeply analyze the influence mechanism of social commerce features on consumer behavior. For example, Hajli (2015) showed that social commerce structures (i.e., recommendations, ratings and reviews, forums, and communities) are essential factors in successful customer retention. Hu et al. (2016) verified that peers’ similarity, expertise, and kindness positively influence perceived value and purchase intention in social shopping websites. However, Lin et al. (2017) reported that social commerce is favored by consumers because it is different from e-commerce in terms of interactivity, recommendations, and feedback. Therefore, based on the above research, we conceptualize the social commerce features into three dimensions: interactivity, recommendations, and feedback.

Interactivity refers to a consumer’s perception of the interactivity level of a seller’s social commerce website, which can capture whether the website is active and whether the seller frequently interacts with his/her followers (Zhang et al., 2016; Lin et al., 2017; Lee et al., 2021). The development of social media breaks the limitations of geographical space and promotes human-to-human communication and information sharing (Bowen et al., 2021). Social commerce, which thrives on social media, provides a new way for consumers to participate deeply in the transaction process and meet shopping expectations (Vazquez et al., 2020). Through the interactivity of social commerce, customers can easily communicate with others during the shopping process and share their own consumption experience, and they also expect others to share their experience (Hajli, 2014). This interactive process is an indispensable part of consumer shopping, enabling consumers to complete shopping tasks while also obtaining a different social experience, enhancing the social attributes of shopping, and making them more willing to participate in social commerce (Qin et al., 2021).

With the development of social media technology, the recommendation system has been successfully applied in many fields, including information retrieval, travel commentary, online learning, e-government, and online shopping, especially in the social shopping aspect of social media platforms (Bunnell et al., 2020). Recommendation is to provide matching choices or product suggestions between friends based on each other’s needs (Lin et al., 2017). Recommendations can be viewed as product or service information delivered through informal communication channels, such as phone calls, emails, online messages, and verbal communication, designed to help consumers fully understand products or services before shopping, and this information may affect their consumption expectations (Senecal and Nantel, 2004). Due to the complexity and technical improvement of products, the content generated by user recommendations provides a large amount of information about the product and enables consumers to quickly understand the target product (Weeks et al., 2021). It also gives unique personal advice to others, which will help consumers make timely and accurate purchase decisions. Chen et al. (2018) found that social commerce users actively look for information about a product they intend to buy and get recommendations and suggestions from other users to gain an in-depth understanding of the product and make decisions based on such information.

Feedback is the product or service information that consumers feedback to sellers, which is the product ratings and evaluations generated by consumers on social networking sites after purchasing and using the product or service (Agag and Eid, 2020). In social media environments, social commerce platforms and other retail sites offer consumers the opportunity to post product feedback that includes digital star ratings (usually, 1–5 stars) and genuine reviews about the product written by customers (Mudambi and Schuff, 2012). Through this feedback, consumers can reduce the risk and uncertainty of buying; merchants can better understand and attract consumers, enhance social interaction, and encourage them to provide advice and suggestions, thus creating a good shopping community environment (Meilatinova, 2021). In addition, research has shown that consumers’ comments on shopping sites can improve consumers’ perception of the usefulness and social presence of the site, attract more potential consumers to browse the site for information, and enhance their loyalty (Kumar and Benbasat, 2006).

This study considers interactivity, recommendations, and feedback to be three critical social commerce features based on the above discussion. They reflect the primary attributes of social commerce and promote user-generated content by enhancing social interaction and information sharing among members, thereby influencing consumers’ shopping decisions (Meilatinova, 2021). Therefore, this study uses interactivity, recommendations, and feedback as environmental stimuli to explore the underlying mechanisms of their impact on consumers’ perceived value and repurchase intention.

### Perceived Value as Consumers’ Internal States (O)

In the SOR theory, the organism (O) represents the changes in personal emotions and internal states caused by environmental stimuli (Sultan et al., 2021). In this study, the organism refers to consumer’s perceived value, which involves consumers’ overall assessment of perceived benefits and perceived costs, and is a personal judgment made by consumers after multi-party trade-offs (Zeithaml, 1988). Perceived benefits refer to benefits (such as price advantage, product usefulness, and pleasant mood) that consumers get from shopping. In contrast, perceived costs generally refer to the expenditure of obtaining products or services (such as time and money; Featherman et al., 2021). Consumers obtain value by assessing benefits and costs, generating appropriate behaviors or intentions (Qi and Ploeger, 2021). This means that enterprises can improve consumers’ perceived value by increasing benefits and reducing costs, thus enhancing the generation of repurchase intention (Lee et al., 2016). Consumers’ sense of perceived value in a social commerce environment is related to their social interaction and information acquisition on the platform. Through interactivity, recommendations, and feedback, consumers can perform shopping tasks with the help of other people’s information sharing and experience emotional pleasure through real-time online communication, which helps them make purchase decisions (Hajli, 2014). Research on consumer behaviors has also confirmed that perceived value significantly affects purchase intention (Hu et al., 2016; Lee and Hwang, 2016).

Previous studies of perceived value have focused on the utilitarian aspects of the shopping experience, described as functional values related to shopping tasks, involving several factors, such as shopping convenience, price, and product information (Zhang et al., 2017; Lin et al., 2020). However, traditional utilitarian explanations ignore the hedonic value of shopping and fail to fully reflect the consumer’s shopping experience (Chiu et al., 2014). Carbone and Haeckel (1994) found that most human behaviors are essentially pleasure-seeking, while online consumers usually hope to obtain pleasure through the service experience. This hedonic value is also considered a critical factor in understanding consumers’ shopping behavior. Deci and Ryan (1985) divided the motivations driving human behavior into two categories: extrinsic and intrinsic motivation. External motivation emphasizes performing behavior to achieve a specific goal or reward, similar to the utilitarian benefits generated by utilitarian value. For example, consumers choose online shopping because they seek convenience, abundant commodities, comprehensive product information, and money-saving benefits (Weeks et al., 2021). Intrinsic motivation refers to the feelings of happiness and satisfaction gained from performing certain behaviors strongly associated with hedonic needs (Chang et al., 2020). This shows that perceived value contains multiple dimensions, and we can classify its dimensions from two perspectives: utilitarian value and hedonic value (PicotmCoupey et al., 2021). Utilitarian value involves consumers’ perceived usefulness of the shopping process and results and assesses the functional role of products or services (Shuiqing et al., 2018). Hedonic value refers to the sense of emotional comfort and satisfaction gained through interaction with peers or other users and feelings of happiness experienced in the interaction process (To et al., 2007). In past research, these two types of perceived value have also been widely applied in consumer behavior research (Dedeoglu et al., 2018; Alzayat and Lee, 2021; Sultan et al., 2021). These two values also significantly influence purchase intentions in social commerce (Lin et al., 2020). Therefore, this study will explore the impact of utilitarian value and hedonic value on social commerce consumers. Utilitarian value refers to consumers’ perceptions of functional benefits, which means that social commerce platforms help consumers complete shopping tasks more conveniently and reduce costs (Hsu and Lin, 2016). Hedonic value is related to the emotional experiences gained from interactions between consumers and others during the shopping process (Van der Heijden, 2004).

### Repurchase Intention as the Response (R)

In the SOR theory, the response (R) refers to the customer’s behavioral response. In the context of online shopping, consumers’ responses to external environmental stimuli are associated with consumption decisions, which are often referred to as behavioral intention (Ma and Wang, 2021). This study uses consumers’ repurchase intention as a behavioral response. Repurchase intention refers to the consumer’s subjective willingness to purchase products multiple times in the same store or seller (Chiu et al., 2014). Compared to potential consumers, repurchasers have a more comprehensive understanding of a seller’s products or services due to their existing purchasing experience, can better evaluate the information and attributes of merchants and products, and make repurchase decisions (Lee and Charles, 2021). Meanwhile, the motivation to repurchase is also different from the motivation to make an initial purchase. According to Liao et al. (2017), website and product attributes, such as website usability, service quality, product price, and merchant reputation, are crucial in forming initial purchase intent. However, their importance to repurchase is significantly lower because consumers hope to obtain more utilitarian value and hedonic value when forming repurchase intentions (Meilatinova, 2021). Moreover, these values are the primary goals that consumers want to achieve when shopping, and they are also the key factors that make the transaction successful (Alzayat and Lee, 2021). In social commerce, perceived value involves the practicability and pleasant shopping experience associated with products or services, as perceived by consumers based on several features, such as interactivity, recommendations, and feedback. Kim and Gupta (2009) also found that perceived value significantly affects consumers’ intentions to repurchase in online shopping. Therefore, this study suggests taking repurchase intention as the response (R), because it can clearly reflect the behavioral response of consumers after the change of their perceived value.

## Research Model and Hypotheses Development

### Social Commerce Features and Perceived Value

Interactivity refers to the extent to which consumers actively participate in social commerce, which facilitates communication between buyers and sellers (Li and Guo, 2021). In meeting the needs of social media users, interactions can usually be divided into two types: One is the interaction with individuals or enterprises in social media; the other is the interaction with social media platforms to evaluate and filter the dynamic information that exists in the platform (Chen, 2017). Gu et al. (2013) called the former interpersonal interaction and the latter human–computer interaction, and they found that both interactions are conducive to realize consumers’ purchase motivation. People mainly participate in two types of interactions through social media and online communities and make use of these interactions to meet their perceived value (Hajli, 2015). Through interaction, consumers can obtain more factual information about product quality and after-sales service and have a more comprehensive and objective understanding of the desired product (Tajvidi et al., 2021). In addition, consumers can also get professional advice, including popular items and coupons, thereby obtaining the optimal purchase method and reducing costs (Wang et al., 2021). Obviously, communication and sharing can help consumers gain convenience and reduce costs in purchasing decisions. Therefore, we propose that:

*H1:* Interactivity is positively associated with utilitarian value.

Consumers can share shopping experiences, give some guidance and advice to sellers, strengthen their connections with each other, and enhance the hedonic value of shopping (Shin and Lee, 2021). Moreover, consumers feel enthusiasm and respect from the seller’s positive response and enjoy the shopping experience (Akram et al., 2021). In previous studies, Kuo and Feng (2013) contend that interactive enable consumers to identify various benefits (e.g., hedonic and learning benefits). Zhang et al. (2014) believe that through the interactivity of social commerce, consumers gain social support, social presence is strengthened, and friendship among community members develops). On balance, the interactivity of social commerce not only meets consumers’ demand for interpersonal communication, but also enables them to obtain psychological satisfaction in the process of supporting and being supported (Berezan et al., 2020). Both of them can significantly improve the value of pleasure perceived by consumers. Based on this, we propose that:

*H2:* Interactivity is positively associated with hedonic value.

Recommendation refers to product information and suggestions that consumers obtain through social networks (Hu et al., 2016). In the network environment, because consumers cannot experience products or services, they rely more on other people’s recommendations (Hajli, 2015; Bhattacharyya and Bose, 2020). These recommendations are crucial to consumers, and they can be quickly generated and disseminated through word of mouth, social sharing, and other ways to enhance consumers’ perceived value (Senecal and Nantel, 2004). With the rapid popularization of the Internet, the amount of information available online has exploded, and obtaining relevant information has become very difficult (Lin et al., 2021). Friends have a better understanding of consumers’ purchasing needs and preferences and can make targeted recommendations, thus reducing the time cost of shopping and improving consumers’ perception of utilitarian value (Yoo et al., 2010). Furthermore, consumers who shop online cannot access physical products. Sharing and recommendations from friends can provide them with more channels to learn about products and sellers, which will help them to shop smoothly (Huang and Benyoucef, 2013). Hence, we propose that:

*H3:* Recommendations are positively associated with utilitarian value.

Information overload hinders consumers in completing their shopping tasks and causes psychological stress and social exhaustion (Arazy et al., 2010). Appropriate recommendations provide consumers with new sources of product information, thus reducing the amount of time consumed and mental stress when searching for helpful information (Chen et al., 2017). Remarkably, compared to blindly browsing the web, the information provided by friends or others is more accurate, more trustworthy, and better able to meet consumers” psychological expectations (Jiménez-Castillo and Sánchez-Fernández, 2019). This recommendation can also reduce consumers’ sense of information overload and enhance psychological relaxation, reflecting the degree of support that consumers receive from others and perceived hedonic value (Gu et al., 2021). Moreover, sharing and recommendation among friends strengthen emotional communication, bring spiritual joy, and enhance hedonic value (Busalim et al., 2021). Obviously, the recommendation can make consumers feel relaxed, get emotional comfort, and enjoy the pleasure of shopping. Therefore, we hypothesize that:

*H4:* Recommendations are positively associated with hedonic value.

Feedback, which can be divided into active and passive feedback, refers to relevant information provided by consumers regarding their purchase and use experience with products or services (Bhandari et al., 2021). Active feedback refers to consumers consulting or describing their consumption experiences when using products or services, while passive feedback refers to companies asking consumers opinions about their products or services (Zhang et al., 2017). Both types of feedback transform one-way product promotion and sales channels into two-way communication and value creation platforms, thus facilitating information sharing and enhancing consumers’ perceived utilitarian value (Kang et al., 2016). Feedback can increase the effective communication between buyers and sellers, quickly understand and meet the demands of consumers, and save shopping costs (Li, 2017). Moreover, when consumers want to buy a product in the social commerce platform, they usually consult the previous product reviews or ask others about their experience and opinions on the product (feedback), so as to fully understand and evaluate the target product (Ringler, 2021). Thus, we suggest that:

*H5:* Feedback is positively associated with utilitarian value.

Consumer feedback through ratings and reviews can provide comprehensive information about products or services to other potential customers (Hajli, 2015). Companies can also use feedback to strengthen their services, correct defects in products, and adopt consumer advice to develop products or services that are more responsive to consumer needs (Torres et al., 2014). Both kinds of feedback will bring help to others, realize the social value of consumers, and enhance their hedonic value (Amblee and Bui, 2014). In addition, through timely responses to consumer feedback, companies can also demonstrate their focus on consumers and support and maintain relationships. This can establish and develop a good interactive environment, reduce conflicts, encourage consumers to express their views and suggestions on products or services, and enhance their goodwill towards shopping platforms and gain more enjoyment value (Agag and Eid, 2020). Based on this, we propose that:

*H6:* Feedback is positively associated with hedonic value.

### Perceived Value and Repurchase Intention

**Figure 1:**
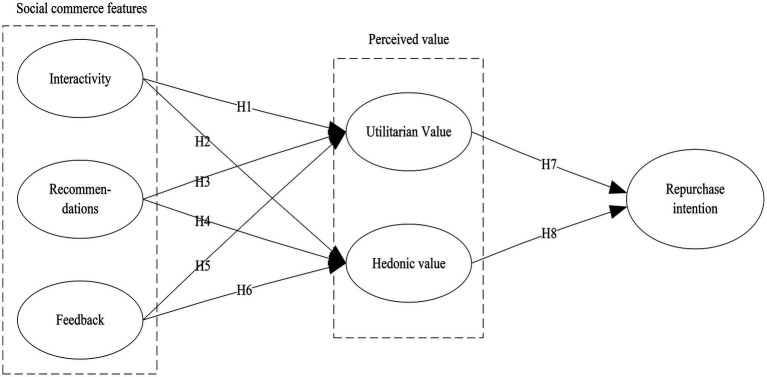
Research model.

Utilitarian value and hedonic value are the cognitive and emotional manifestations of the consumers’ shopping experience (Dedeoglu et al., 2018). When users can quickly and easily find their favorite products through social commerce platforms or feel that the products are worthwhile, they will realize more utilitarian value, experience higher satisfaction with the platform, and generate purchase intentions (Alzayat and Lee, 2021). Furthermore, when users like the shopping process or have positive experiences while using social commerce platforms, they are more satisfied and feel more excellent hedonic value; hence, their buying intentions are strengthened (Tafesse, 2021). Gan and Wang (2017) found that value is the crucial factor in purchase intention, and the more cost consumers save when purchasing products or services, the higher the perceived value and their repurchase intention are more substantial. Moreover, consumers will try their best to realize the utilitarian value and hedonistic value and choose products more in line with their value goals by comparing different platforms and products (Bridges and Florsheim, 2008). Therefore, when products in social commerce can provide higher utilitarian value and hedonic value, consumers will have a stronger desire to repurchase. Previous studies have also confirmed the impact of these two values on purchase intentions. For example, Lee et al. (2016) examined the positive impacts of utilitarian value and hedonic value on social media users’ participation in group buying. Chiu et al. (2014) explored the significant impact of consumers’ utilitarian value and hedonic value on repurchase intention. Consequently, we believe that the utilitarian value and hedonic value will influence consumers’ repurchase intention (Figure 1):

*H7:* Utilitarian value is positively associated with repurchase intention.

*H8:* Hedonic value is positively associated with repurchase intention.

## Research Design

### Scale Development

The questionnaire with five-point Likert scales was used to collect data regarding the demographic characteristics of respondents and research variables. All items were adapted from existing studies. For example, items designed to measure interactivity (IA) were adapted from a study conducted by Zhang et al. (2016); measurements of recommendations (RD) were adapted from Hajli (2015), and feedback (FB) was measured based on Yi and Gong (2013). Items to measure utilitarian value and hedonic value were adapted from a study conducted by Chen and Fu (2018); the measurement of repurchase intention was based on Lin et al. (2017). Since the survey object is Chinese consumers, the English items need to be translated into Chinese, and then, we translated the Chinese items back into English. Next, compare these two different versions of the English items to guarantee that the Chinese scale truly expresses the meaning of the original items. Then, a pilot was conducted with 20 college students. Based on the results of the pilot, we further refined the items to maximize clarity, resulting in the final scale (Supplementary Appendix A).

### Data Collection

We used WeChat as the survey environment for social commerce platforms, and the respondents were consumers who had experienced shopping on WeChat. Since its launch in January 2011, WeChat has grown into China’s most extensive instant messaging application, and its user penetration rate continues to rise. In the first quarter of 2021, WeChat had over 1.24 billion monthly active users, accounting for more than 80% of China’s population (Statista, 2021). Moreover, with the development of Internet technology, online shopping has become increasingly popular among consumers, and the popularity of WeChat has made social media shopping the norm. As China’s most popular instant messaging application, WeChat allows users to create a WeChat official account and use it to display and sell their products and services, and consumers can also browse and purchase goods through WeChat (Chen et al., 2018). Furthermore, WeChat, with its succinct functional interface and operation flow, enables users to quickly and efficiently interact through various formats, such as text, instant voice, music, and video, which is attractive to potential users (Chen et al., 2021). Furthermore, WeChat’s functions, such as “shake,” “drift bottle,” “friend circle,” and “public number,” as well as its emerging Mini Programs, enhance communication between users and meet the social needs of different types of people (Li et al., 2019). WeChat also provides access to all-encompassing product information and enables consumers to experience social commerce features (interactivity, recommendations, and feedback). Therefore, we collect data from consumers who have purchase experience on WeChat.

Our questionnaire is published on the professional online survey platform sojump^1^ in China, and the large user base of this platform can help us survey a broader range of respondents. Through the sample service of this platform, we received a total of 574 completed questionnaires. After deleting the invalid questionnaires, we obtained 514 valid questionnaires. Therefore, the recovery rate of valid questionnaires was 89.547%. In this survey, the proportion of male respondents was 41.634, and 58.366% were female; most were between 20 and 39 years old (86.965%). Furthermore, 82.879% held a bachelor’s degree or above, and the average monthly income level of respondents was above 3,000 yuan (92.607%). Specific statistical characteristics of the sample are presented in Table 2.

**Table 2:** Sample statistical characteristics (*N* = 514).

Attributes	Options	Frequency	Percentage (%)
Gender	Male	214	41.634
	Female	300	58.366
Age	≤19	4	0.778
	20–29	218	42.412
	30–39	229	44.553
	40–49	49	9.533
	50–59	13	2.529
	≥60	1	0.195
Education	Junior high school	4	0.778
	High school	25	4.864
	Associate degree	59	11.479
	Bachelor’s degree	388	75.486
	Master’s degree or higher	38	7.393
Average monthly income (RMB)	<1,000	5	0.973
	1,000–3,000	33	6.420
	3,000–5,000	134	26.070
	5,000–7,000	143	27.821
	7,000–9,000	104	20.233
	>9,000	95	18.482

## Data Analysis

### Common Method Bias Test

Since self-reported questionnaires were used to collect data in this study, there was a risk of introducing common method bias. Therefore, two steps were used to test common method bias. First, we executed Harman’s single-factor test (Podsakoff et al., 2003), and the results of the unrotated exploratory factor analysis informed that the largest factor accounted for 26.867% of the total variance, less than 40%. Second, we examine common method bias through the marker variable method (Malhotra et al., 2006), using a variable (education of respondents) that is theoretically independent of the endogenous variable as the marker variable. The analysis results indicated that the average correlation coefficient between the marker variable and the main variables in our study was 0.029, less than 0.100. Therefore, from these results, we can conclude that the data and tests of this study are unlikely to be affected by common method bias.

### Confirmatory Factor Analysis

We first tested the reliability and validity of the scale. The results showed that the Cronbach’s *α* and composite reliability (CR) of all variables exceeded 0.70 (see Table 3), which indicates that the scale has good reliability and internal consistency. Second, we examine the convergence validity of the variables by calculating the factor loadings and AVE (average variance extracted) value. The results show that the standard loadings of IA3, UV4, HV3, and HV4 were lower than the recommended loading of 0.6, which affected the overall validity of the scale. Therefore, IA3, UV4, HV3, and HV4 were excluded. After eliminating these four items, the factor load values of all the measured items were greater than 0.50 with a range of 0.616–0.831, and the AVE of variables ranged from 0.512 to 0.621, which indicates that the convergent validity of the clean scales is good. Third, discriminant validity was proved by the results that the square root of the AVE value of each variable is greater than the correlation coefficient between the variable and other variables (see Table 4).

**Table 3:** Results of reliability and convergent validity analysis.

Factor	Item	Standard loading	VIF	Weight	CR	Cronbach’s *α*	AVE
Interactivity (IA)	IA1	0.831	1.063	0.691	0.765	0.703	0.621
	IA2	0.742	1.063	0.574			
Recommendations (RD)	RD1	0.678	1.295	0.286	0.811	0.718	0.518
	RD2	0.752	1.346	0.370			
	RD3	0.783	1.381	0.400			
	RD4	0.660	1.200	0.326			
Feedback (FB)	FB1	0.616	1.050	0.445	0.713	0.709	0.512
	FB2	0.730	1.060	0.565			
	FB3	0.671	1.076	0.467			
Utilitarian value (UV)	UV1	0.752	1.291	0.398	0.809	0.711	0.514
	UV2	0.681	1.248	0.317			
	UV3	0.702	1.264	0.338			
	UV5	0.731	1.330	0.339			
Hedonic value (HV)	HV1	0.749	1.387	0.339	0.831	0.729	0.552
	HV2	0.691	1.291	0.311			
	HV5	0.765	1.428	0.347			
	HV6	0.763	1.422	0.348			
Repurchase intention (RI)	RI1	0.758	1.226	0.445	0.808	0.710	0.584
	RI2	0.783	1.314	0.434			
	RI3	0.752	1.258	0.429			

* *p* < 0.05.

** *p* < 0.01.

*** *p* < 0.001.

**Table 4:** Results of discriminant validity analysis.

Factor	IA	HV	UV	FB	RD	RI
IA	**0.788**					
HV	0.382	**0.743**				
UV	0.419	0.619	**0.717**			
FB	0.354	0.268	0.389	**0.716**		
RD	0.309	0.480	0.377	0.176	**0.720**	
RI	0.404	0.584	0.661	0.414	0.417	**0.764**

Diagonally arranged values (the bold values) are the square roots of average variance extracted (AVE); off-diagonal values represent the correlation coefficients.

In addition, we also performed a multicollinearity test, and the variance inflation factor (VIF) value, weight, and significance level of all structures were evaluated. The results show that the VIF values of all structures are lower than 10, indicating that there is no problem with multicollinearity in our study.

### Hypothesis Testing

**Figure 2:**
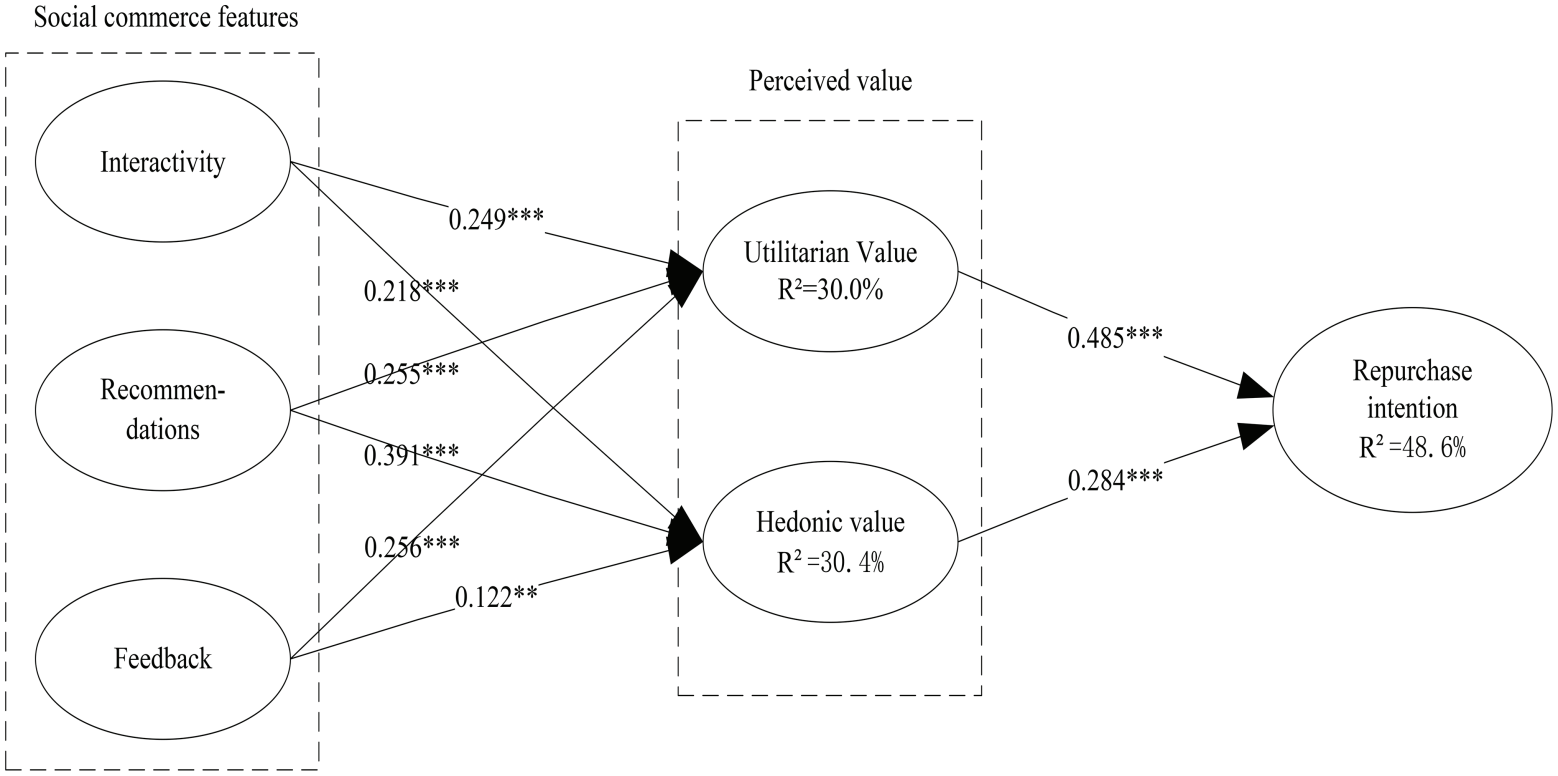
Hypothesis testing results (^*^p < 0.05; ^**^p < 0.01; ^***^p < 0.001).

We used SmartPLS to test the hypothesis, and this technique was selected for two reasons: (1) the PLS (partial least squares) technique has been widely used in IS research, which can test the precise model fit and be used for exploratory theory building (Braojos et al., 2020). This study utilizes an emerging research model to explore the effects of social commerce features and perceived value on consumers’ repurchase intention. (2) PLS has advantages in model estimation of small to medium sample sizes (Yu et al., 2018). In this research, our sample size of 514 is not large, which is sufficient for the use of the PLS technique. (3) PLS does not require identical distribution of residuals. Hence, we believe that SmartPLS is a suitable technique. All the results are shown in Figure 2. We found that interactivity has positive effects on utilitarian value (*β* = 0.249, *p* < 0.001) and hedonic value (*β* = 0.218, *p* < 0.001), thus supporting H1 and H2. Recommendations positively affect utilitarian value (*β* = 0.255, *p* < 0.001) and hedonic value (*β* = 0.391, *p* < 0.001), which means that H3 and H4 were supported. Feedback positively affects utilitarian value (*β* = 0.256, *p* < 0.001) and hedonic value (*β* = 0.122, *p* < 0.01), indicating that H5 and H6 were supported. Utilitarian value (*β* = 0.485, *p* < 0.001) and hedonic value (*β* = 0.284, *p* < 0.001) have positive effects on repurchase intention, thus supporting H7 and H8. Therefore, all the hypotheses of this study are valid and have high significance. The explained variances for utilitarian value, hedonic value, and repurchase intention are 30.0, 30.4, and 48.6%, respectively. This finding indicates that consumers can clearly perceive the benefits brought by the platform through interactivity, recommendations, and feedback, thus forming utilitarian value and hedonic value. Utilitarian value and hedonic value also contribute substantially to the formation of repurchase intention. In addition, this study also examined the influence of control variables such as gender and age on repurchase intention. Among them, gender significantly affects repurchase intention, which is consistent with previous studies on social commerce (Xuequn et al., 2019; Molinillo et al., 2021), and they found that gender differences affect consumer behavior in social commerce.

### Test of Mediating Effects

The model implies mediating effects. Utilitarian value and hedonic value are mediating variables that affect repurchase intention through social commerce features. Therefore, to evaluate the mediating effect of utilitarian value and hedonic value, we tested for mediation effects by applying the steps recommended by Anthony et al. (2015) using 5,000 bootstrap samples with 95% confidence intervals (CIs). First, we examined the impact of mediating variables through three paths: (1) the impact of independent variables on mediating variables (path a), (2) the impact of mediating variables on dependent variables (path b), and (3) the path from independent variables to dependent variables (path c or c′ when both paths a and b are considered). Next, we determine whether a full or partial mediation occurred by examining the CI for c′. If ab is nonzero and c′ is zero, it means full mediating effect. If both ab and c′ are nonzero, a partial mediating effect is indicated (Shrout and Niall, 2002). Test results of mediating effect are given in Table 5. The conclusion is that interactivity, recommendations, and feedback not only indirectly affect repurchase intention through utilitarian value and hedonic value but also directly affect repurchase intention (i.e., there is a partial mediating effect).

**Table 5:** Mediating effects tests.

IV	M	Mediation test (ab)			Full/Partial mediation test (c′)			Type of mediation
		2.5% lower bound	97.5% upper bound	Zero included?	2.5% lower bound	97.5% upper bound	Zero included?	
IA	UV	0.122	0.222	NO	0.034	0.160	NO	Partial
IA	HV	0.055	0.137	NO	0.034	0.160	NO	Partial
RD	UV	0.123	0.235	NO	0.068	0.212	NO	Partial
RD	HV	0.061	0.169	NO	0.068	0.212	NO	Partial
FB	UV	0.132	0.256	NO	0.133	0.288	NO	Partial
FB	HV	0.051	0.128	NO	0.133	0.288	NO	Partial

IV, independent variable; M, mediator. a, b, c and c′ are path coefficients, respectively.

## Discussion and Implications

### Discussion of Results

From the theoretical perspective of the SOR theory and perceived value, we explore the factors that influence the formation of consumers’ willingness to repurchase on social commerce platforms. Data were collected *via* a questionnaire, and empirical analyses were performed to validate the measurement model of this study. The findings confirm our hypotheses, and the specific results are as follows:

First, social commerce features significantly affect consumers’ perceived value. Our hypothesis test results suggest that interactivity, recommendations, and feedback have positive effects on consumers’ perception of utilitarian value and hedonic value, and the variance interpretation rates are 30.0 and 30.4%, respectively. This shows that the shopping experience in social commerce makes consumers perceive sufficient utilitarian value and hedonic value, which subsequently stimulates their intention to repurchase. And in this process, the recommendations have a more significant impact than interactivity and feedback, which may be due to consumers not being able to obtain enough information about products or services while shopping, but the pressure to complete shopping tasks and make the right decisions, they spend a lot of time searching for the correct information to reduce the uncertainty of shopping. Thus, recommendations in social commerce help meet consumers’ needs, helping them filter available information, save time on shopping, and make better decisions. In addition, the impact of feedback is relatively minimal, which suggests that consumers pay more attention to information exchange and sharing with others before shopping and obtain product recommendation information from user-generated content generated by interaction.

Second, as two crucial dimensions of perceived value, utilitarian value and hedonic value significantly impact repurchase intention. This result is consistent with previous research on consumer perceived value (Peng et al., 2019; Wang et al., 2019); that is, the higher the perceived value of a product, the stronger the intention of consumers to buy it. We also found that consumers are more concerned about utilitarian value, and utilitarian value is more likely to affect their repurchase intention than hedonic value. This result expands previous research on e-commerce (Chiu et al., 2014), reflecting the importance of utilitarian value in predicting consumers’ repurchase intention. At the same time, this also means that compared to the emotional pleasure that a social commerce platform can bring, consumers pay more attention to the platform’s helpfulness in achieving their shopping goals; thus, the benefits generated by information exchange among users can better improve consumers’ perceived value.

Third, after performing mediating effects testing, we found that perceived value is a mediator between social commerce features and repurchase intention. This extends the previous research on the mediating role of perceived value (Ozturk et al., 2016) and further verifies the crucial role of perceived value in promoting consumers’ purchase intention. Specifically, interactivity, recommendations, and feedback are the initial experience of consumers’ participation in social commerce, and the goal of these features is to make consumers feel more utilitarian value and hedonic value. After that, under the incentive of this high value, consumers are encouraged to have a corresponding repurchase intention. This reveals the critical role of social commerce features and perceived value in consumers’ repurchase intentions and enriches the theoretical research on individual behavior in the existing social commerce environment.

### Theoretical Implications

This study has two main contributions to social commerce and individual behavior research. First, in previous studies, social commerce features and consumers’ perceived value are often considered as antecedent variables of purchase intention, and their direct effects on purchase intention are considered (Aspara et al., 2021; Zhou et al., 2021). Few studies have explored the effect of social commerce features on purchase intention from the perspective of perceived value and examined the mediating effect of perceived value (Mou and Benyoucef, 2021). Based on SOR theory, this study constructed the influencing factors model of consumers’ repurchase intention by taking social commerce features as the stimulus factor, perceived value as the internal state, and consumers’ repurchase intention as the final response. By analyzing how consumers form repurchase intention in the social commerce environment, this study clarifies the influence mechanism of social commerce features on repurchase intention, provides new empirical evidence for understanding the important role of social commerce features and enriches the existing literature on social commerce.

Second, for the platform to continue to develop, it needs to listen carefully to the needs of consumers and improve their intention to continue to participate or repurchase. Given this, we carried out a deep analysis of repurchase intention, clarified its pivotal role in social commerce platforms, and identified multiple factors affecting consumers’ repurchase intention. It helps fill the gaps in the research on repurchase intention and provides a reference for further analysis of its influencing factors. In addition, this study also discusses the influence of utilitarian value and hedonic value on consumers’ repurchase intention and verifies the mediating role of the two. This further highlights the importance of perceived value, indicating that utilitarian value and hedonic value can significantly predict consumer behavioral responses. The more value consumers perceive from social commerce, the stronger their intention to repurchase.

### Managerial Implications

Based on theoretical research and the empirical analyses of our research model, the findings have some management implications. First, we can see from the results that social commerce features significantly affect perceived value. Therefore, we should strengthen the influence of interactivity, recommendations, and feedback on consumers to increase their perceived benefits and value. For example, merchants should create a more convenient communication channel to promote real-time and diverse information exchange between users and focus on stimulating and maintaining activity in this community, thus improving the user’s interactive experience. Furthermore, businesses should also develop appropriate rules to regulate user communication and create a harmonious and healthy communication environment while protecting users’ privacy and preventing information leakage. For recommendations and feedback on social commerce platforms, merchants can analyze users’ consumption preferences according to their browsing and purchasing records and make full use of big data technology to provide personalized product recommendations for users and promote purchase intentions. Merchants should also improve the relevant service level to provide comprehensive after-sales services and address consumers’ questions or requirements for products or services promptly. Through cash or another type of reward, merchants should encourage consumers to share their shopping experience and inspire them to express their suggestions or opinions so as to integrate feedbacks from various parties to improve the quality of products or services.

Second, because utilitarian value and hedonic value can promote the generation of repurchase intention, merchants or enterprises should strengthen consumers’ perceived utilitarian value and hedonic value through various ways to enhance their willingness to purchase. In view of the practicability of products or services involved in utilitarian value, merchants should fully optimize the platform’s layout, functional interface, and operational processes of social commerce so that consumers can use the platform quickly and easily for shopping. Merchants should also strengthen the impact of their low-price strategies on consumers, carry out product promotion activities from time to time, and ensure that consumers get real discounts. To increase consumers’ hedonic value derived from shopping, sellers should simplify the search process for product information and highlight product recommendations and feedback that consumers care about to minimize information overload, which would help customers understand the products more fully and make decisions more quickly. Moreover, it is necessary to promptly deal with a series of issues about products and services provided by consumers and encourage other platform users to actively participate in product Q&A to provide information support and emotional support, which enhances consumers’ perceived hedonic value.

### Limitations and Recommendations

Although the study draws some important conclusions, several limitations are noteworthy. First, the research object of this paper is WeChat users who have a social shopping experience. This approach is highly targeted and does consider other social media. Because the perceived value of consumers on different platforms may also be different, future research should expand the scope of the research object, consider differences between platforms, and enhance the applicability of the research results. Second, our structural model explains 48.6% of the variance in repurchase intention, indicating that other relevant factors did not take complete account. Future research can consider factors influencing repurchase intention from a broader perspective. Third, consumers’ online shopping experiences involve the platform’s features and related product features, such as product price and quality. Therefore, future research models should incorporate specific product features simultaneously. Fourth, for reflecting the uniqueness of social commerce, future research should further explore the features of social commerce to enrich the research of social commerce.

## Data Availability Statement

The raw data supporting the conclusions of this article will be made available by the authors, without undue reservation.

## Author Contributions

All authors listed have made a substantial, direct and intellectual contribution to the work, and approved it for publication.

## Conflict of Interest

The authors declare that the research was conducted in the absence of any commercial or financial relationships that could be construed as a potential conflict of interest.

## Publisher’s Note

All claims expressed in this article are solely those of the authors and do not necessarily represent those of their affiliated organizations, or those of the publisher, the editors and the reviewers. Any product that may be evaluated in this article, or claim that may be made by its manufacturer, is not guaranteed or endorsed by the publisher.

## Footnotes

^1^ www.sojump.com
