# Supplementary material for: Exploring the Relationship Between Social Commerce Features and Consumers’ Repurchase Intentions: The Mediating Role of Perceived Value
Source: Front Psychol. 2022 Mar 25;12:775056. doi: 10.3389/fpsyg.2021.775056 (PMC8990309; doi:10.3389/fpsyg.2021.775056)
Supplement: Supplementary file 1 [file Data_Sheet_1.docx]

Supplementary Material

**Appendix A: Measurement Scales**

| Variable | Item | Content | Source |
| --- | --- | --- | --- |
| Interactivity（IA） | IA1 | Sellers can actively communicate with me on WeChat. | Zhang et al. (2016) |
|  | IA2 | Sellers often interact with me on WeChat. |  |
|  | IA3 | Sellers can respond promptly to my inquiries or comments on WeChat. |  |
| Recommendations (RD) | RD1 | I think the suggestions made by friends on WeChat are generally frank. | Hajli (2015) |
|  | RD2 | I think the suggestions of friends on WeChat are generally reliable. |  |
|  | RD3 | In general, the suggestions of friends on WeChat are trustworthy. |  |
|  | RD4 | I trust my friends on WeChat and share my status and photos with them. |  |
| Feedback (FB) | FB1 | If I have a useful idea of how to improve a product or service, I will let sellers know. | Yi and Gong (2013) |
|  | FB2 | When I receive premium products or services from sellers, I will comment on them. |  |
|  | FB3 | When I encounter a problem, I will let sellers know. |  |
| Utilitarian value (UV) | UV1 | WeChat enhances my effectiveness in buying organic foods. | Chen and Fu (2018) |
|  | UV2 | WeChat enables me to complete the task of purchasing organic foods faster. |  |
|  | UV3 | WeChat enables me to complete the task of buying organic foods anytime and anywhere. |  |
|  | UV4 | WeChat offers a variety of functions that allow me to complete different tasks for purchasing organic foods (delete) |  |
|  | UV5 | In general, WeChat has improved my efficiency in buying organic foods. |  |
| Hedonic value (HV) | HV1 | Buying organic foods on WeChat makes me feel relaxed. | Chen and Fu (2018) |
|  | HV2 | Buying organic foods on WeChat allows me to keep up with fashion trends. |  |
|  | HV3 | Buying organic foods on WeChat makes me feel new (delete). |  |
|  | HV4 | I usually immerse myself in WeChat for hours (delete). |  |
|  | HV5 | Buying organic foods on WeChat makes me feel satisfied. |  |
|  | HV6 | Buying organic foods on WeChat makes me very happy. |  |
| Repurchase intentions (RI) | RI1 | I will consider purchasing organic foods from sellers on WeChat in the near future. | Lin et al. (2017) |
|  | RI2 | I am planning to place an order with organic food sellers on WeChat again. |  |
|  | RI3 | I will buy similar organic foods from sellers on WeChat again. |  |
